# Association analysis of *RTEL1* variants with risk of adult gliomas in a Korean population

**DOI:** 10.1371/journal.pone.0207660

**Published:** 2018-11-21

**Authors:** Suhg Namgoong, Hyun Sub Cheong, Jeong-Hyun Kim, Lyoung Hyo Kim, Jung Yeon Seo, Seok-Gu Kang, Seon-Jin Yoon, Se Hoon Kim, Jong Hee Chang, Hyoung Doo Shin

**Affiliations:** 1 Department of Life Science, Sogang University, Seoul, Republic of Korea; 2 Department of Genetic Epidemiology, SNP Genetics Inc., Seoul, Republic of Korea; 3 Asan Institute for Life Sciences, University of Ulsan Collage of Medicine, Seoul, Republic of Korea; 4 Department of Neurosurgery, Yonsei University College of Medicine, Seoul, Republic of Korea; 5 Department of Biochemistry and Molecular Biology, College of Medicine, Yonsei University, Seoul, Republic of Korea; 6 Department of Pathology, Yonsei University College of Medicine, Seoul, Republic of Korea; 7 Research Institute for Basic Science, Sogang University, Seoul, Republic of Korea; University of Texas Rio Grande Valley, UNITED STATES

## Abstract

Previous studies have identified multiple loci for inherited susceptibility to glioma development, including the regulator of telomere elongation helicase 1 (*RTEL1*). However, the association between *RTEL1* variants and risk of glioma has not been well understood. Therefore, we sought to comprehensively examine the genetic interaction between *RTEL1* variants and risk of glioma with respect to defined histological and molecular subtypes. We employed a case-control study involving 250 adult glioma patients with previous molecular alterations and 375 population–based controls within Korean populations. Statistical analyses on the association between *RTEL1* single nucleotide polymorphisms (SNPs) and glioma risk were conducted using unconditional logistic regression. Additional conditional and stepwise analyses were performed on significant *RTEL1* SNPs. We detected significant associations (Bonferroni *P* < .05) between six SNPs (*rs6089953*, *rs3848669*, *rs6010620*, *rs3787089*, *rs6062302*, and *rs115303435*) and risk of glioma in the Korean subjects. The two coding variants, *rs6062302* (D664D) and *rs115303435* (A1059T), were plausibly causal variants and were independent among the significantly associated *RTEL1* variants. The glioma subgroup analyses showed that the causal variants (*rs6062302* and *rs115303435*) may be associated with increased risk of glioma regardless of histological grades and molecular alterations. This study provides a deeper understanding of relationships between *RTEL1* variants and risk of glioma. Further studies are required to ascertain the impact of those variants on glioma susceptibility.

## Introduction

Glioma is a common tumor which develops within the central nervous system (CNS) [1, 2]. It is derived from of the neuroglial stem and progenitor cells, accounting for 28% of all brain primary tumors and 80% of malignant brain tumors [2]. With the implementation of 2016 World Health Organization Classification of Tumors of the CNS (2016 CNS WHO), the gliomas of the brain are required to be diagnosed with the isocitrate dehydrogenase (*IDH*) and the chromosome abnormalities of 1p and 19q status [3]. These genetic alterations have been accepted by worldwide neuro-oncology groups, and most of the glioma patients are being classified into the category of diffuse astrocytoma, anaplastic astrocytoma, oligodendroglioma, anaplastic oligodendroglioma, or glioblastoma (GBM) with the molecular signatures [3–5].

In addition to the integrated phenotypic and genotypic features of glioma, many genetic studies have found that common inherited variants near several genes (*TERC*, *TERT*, *EGFR*, *CDKN2B*, *PHLDB1* and *RTEL1*) are associated with increased risk of adult glioma [6]. Genome-wide association studies (GWASs) have found that variants in the regulator of the telomere elongation helicase1 (*RTEL1*) gene are associated with increased risk of adult glioma in White populations [7–9]. In case-control studies for glioma, the associations were detected for the intronic SNPs (*rs6010620* and *rs2297440*) of *RTEL1* in the United States (US) [10] and Han Chinese populations [11]. Meta-analyses showed that *rs6010620* is associated with increased risk of glioma in populations of both European and Asian descent, although this SNP has an inconclusive effect on glioma risk [12, 13]. In molecular groups of gliomas that have gained the telomerase reverse transcriptase (*TERT*) promoter mutation, *IDH* mutation, and 1p/19q codeletion, *rs6010620* serves as protection against glioma susceptibility in *TERT* mutation status [14].

However, association between *RTEL1* SNPs, including coding variants, and risk of gliomas is not obvious. Therefore, we analyzed the selected *RTEL1* SNPs, including previous glioma variants, for association with risk of adult gliomas in Korean populations. We also examined the possible interactions between susceptibility alleles and glioma subgroups such as grades, histologic features, and molecular information.

## Materials and methods

### Study subjects

The blood samples of 250 Korean glioma patients were collected at the Yonsei University Severance Hospital and collaborating hospitals, diagnosed between 2006 and 2016. Case subjects were older than 18 years of age and were categorized into glioma subtypes based on histopathological and molecular features according to the 2007 and 2016 WHO classification of CNS tumors [3]. For molecular alterations, *IDH1* or *IDH2* mutation and 1p/19q codeletion status were observed using previously described methods [15, 16]. The institutional review board of Yonsei University Severance Hospital approved the study protocols and the patients gave written informed consent for participation. As controls, a total of 375 unrelated population-controls (PCs), which excluded participants who had past medical history of various cancer types, were collected from the National Biobank of Korea, the Korean Genome and Epidemiology Study (KoGES) Consortium [17]. The PCs consisted of quality-controlled biospecimen collections from population-based cohorts which comprised 10,038 blood donors aged 40 to 60 years from the Ansung-Ansan Community-based Cohort in 2001. Genomic DNA of blood samples was isolated using the Wizard Genomic DNA Purification Kit (Promega, Madison, WI) for genetic analyses.

### SNP selection and genotyping

The candidate *RTEL1* SNPs were filtered to remove those sites with minor allele frequency (MAF) <5% in Han Chinese Beijing and Japanese Tokyo panels from the 1000 Genomes Project [18]. The SNPs (*rs6089953*, *rs6010620*, *rs4809324*, *rs6062302*, and *rs3208008*) were included according to previous associations with risk of gliomas [9, 10]. The final 26 SNPs in *RTEL1* were selected on the basis of high linkage disequilibrium (LD) between SNPs of interest (*r*^*2*^ >.98). The glioma SNP *rs2297440* was excluded from our study because of perfect LD (*r*^*2*^ = 1) with *rs6010620* in East Asian populations [18, 19]. We also included low frequency (MAF ≤5%) of seven non-synonymous SNPs (*rs184051277*, *rs199685200*, *rs77086616*, *rs199796539*, *rs200933423*, *rs115303435*, and *rs115264605*), except that non-synonymous SNPs were not designable on Fluidigm SNP Type assays (Fluidigm Corp., South San Francisco, CA, US). In addition, all loci were genotyped by the Fluidigm high-throughput platform and Fluidigm EP1 SNP Genotyping 192.24 Dynamic Array. The discrete genotype data were analyzed with the BioMark SNP Genotyping analysis software (version 4.3.2). Among 26 *RTEL1* SNPs, four SNPs (*rs184051277*, *rs199685200*, *rs199796539*, and *rs115264605*) were monomorphic and excluded from additional statistical analysis.

### Statistics

For each SNPs, the deviation of the genotype proportions from those expected under Hardy-Weinberg equilibrium (HWE) was examined. LD analysis between genotyped SNPs was carried out using the Haploview v4.2 software from the Broad Institute (http://www.broadinstitute.org/mpg/haploview). Haplotypes of each individual were reconstructed using the PHASE 2.1 software [20]. Odds ratios (ORs) and corresponding *P*-values were calculated using unconditional logistic regression under an additive model, adjusted for age and sex as covariates. The significant *P*-values were corrected for multiple testing of 22 times using Bonferroni correction. In addition, conditional logistic regression and stepwise analysis were undertaken to identify the independence of association among the significant *RTEL1* variants. All statistical analyses were conducted using SAS 9.4 software (SAS Inc., Cary, NC, US).

### Functional analysis

To predict the function of significantly associated *RTEL1* SNPs with risk of glioma, *in silico* analysis for non-synonymous SNPs was performed using TransFIC (http://bbglab.irbbarcelona.org/transfic/home), which includes well-known tools (SIFT, Polyphen2, MutationAssessor) for assessing the impact of variants in cancer. The TransFIC method normalizes the results from the tools on a baseline tolerance of missense SNPs with dissimilar functions. FuncPred (https://snpinfo.niehs.nih.gov/snpinfo/snpfunc.html) was used to identify the transcriptional regulation of significant *RTEL1* SNPs.

## Results

### Subjects characteristics

All glioma patients composed of astrocytic and oligodendroglial tumor groups fulfilled the inclusion criteria defined by the 2007 WHO classification [21]. The majority of gliomas (92%) which included test results of *IDH* mutation and 1p/19q codeletion status were categorized as astrocytomas, oligodendrogliomas, and GBM as defined by the 2016 CNS WHO classification [3]. The *IDH*-mutants were found in 72 (29.6%) of the 243 glioma patients and the rate of glioma patients with 1p/19q codeletion was 25.9% (61 of 236). Molecular alterations in GBM were less prevalent in both *IDH* mutants (10.9%) and 1p/19q codeletions (11.6%). The case group consisted of patients with 250 unrelated adult gliomas (mean age 51.13 ± 14.71, 52.4% male) and the control group consisted of 375 individuals over 40 years old (mean age 53.61 ± 8.97, 46.9% male). The detailed histologic and acquired molecular features of cases are summarized in Table 1. In addition, we compare molecular characteristics of obtained *IDH* mutations and 1p/19q codeletions with other glioma studies (S1 Table).

**Table 1:** Clinical characteristics of study subjects.

Groups (WHO grade)	Number of subjects	Age (Mean±SD)	Male (%)	Molecular alteration (n)	
				*IDH1 or IDH2* status (mutant /wildtype /NOS)	1p/19q codeletion (yes /no /NOS)
Gliomas (II–IV)	250	51.1±14.7	52.4%	72/171/7	61/175/14
Diffuse astrocytoma (II)	28	46.5±13.1	50.0%	16/12/0	6/22/0
Anaplastic astrocytoma (III)	29	41.1±14.4	48.3%	8/20/1	4/23/2
Oligodendroglioma (II)	16	46.1±7.5	50.0%	14/0/2	16/0/0
Anaplastic oligodendroglioma (III)	18	43.6±11.2	61.1%	17/0/1	18/0/0
Glioblastoma (IV)	159	55.1±14.4	52.8%	17/139/3	17/130/12
Population controls	375	53.6±9.0	46.9%	-	-

Abbreviation: SD, standard deviation; *IDH*, isocitrate dehydrogenase; NOS, not otherwise specified genetic testing of gliomas.

### Genotyping results and associations between *RTEL1* SNPs and glioma risk

The genotyped SNPs’ location of *RTEL1* is displayed in S1A Fig. Three LD blocks were constructed from the 22 *RTEL1* SNPs (S1B and S2 Figs). We found that the four sets of SNPs (*rs6089953*-*rs3848669*, *rs6010620*-*rs3787089*, *rs62207047*-*rs4809324*, and *rs3208008*-*rs2297441*) had high LD (*r*^*2*^ >.90). Although the genotype distributions for SNPs were in accordance with HWE (*P* >.05), a novel missense variant (*rs77086616*, T434M) in *RTEL1* was only observed in anaplastic astrocytoma and GBM patients (HWE *P* = 1.55x10^-16^). In addition, 13 *RTEL1* SNPs were found to have significant associations with risk of adult gliomas in Korean populations (Table 2). After applying the of Bonferroni correction (threshold *P* = .0023), four intronic (*rs6089953*, *rs3848669*, *rs6010620*, *rs3787089*), one synonymous (*rs6062302*, D664D), and one missense (*rs115303435*, A1059T) SNP were found to be significant for glioma risk.

**Table 2:** Genotyped *RTEL1* SNP information and associations of variants with risk of glioma.

SNP (allele)	Chr. position (GRCh38.p7)	SNP location (amino acid change)	Minor allele frequency			HWE *P*			OR (95% CI)	*P*	*P*^*corr*^^b^
			Patients (n = 250)	PCs (n = 375)	Total (n = 625)	Patients (n = 250)	PCs (n = 375)	Total (n = 625)			
*rs6089759* (T>G)	63656966	Promoter	.166	.115	.135	.388	.586	.885	1.61 (1.15–2.25)	**.005**	NS
*rs6122022* (C>A)	63658242	5'UTR	.078	.076	.077	.647	.111	.130	1.02 (0.65–1.58)	.93	NS
*rs2297432* (C>T)	63659310	5'UTR	.150	.124	.134	.852	.557	.556	1.25 (0.90–1.74)	.17	NS
*rs6089953*^a^ (A>G)	63659655	Intron 2	.316	.240	.270	.386	.497	.951	1.52 (1.17–1.97)	**.001**	**.03**
*rs2738778* (C>T)	63660477	Intron 2	.454	.493	.478	.008	.500	.233	0.82 (0.66–1.04)	.10	NS
*rs3848669* (G>T)	63669458	Intron 8	.316	.237	.269	.386	.592	.974	1.54 (1.19–2.01)	**.0009**	**.02**
*rs35902944* (G>C)	63677042	Intron 10	.365	.324	.340	.415	.786	.710	1.19 (0.93–1.51)	.15	NS
*rs34538116* (C>T)	63677077	Intron 10	.320	.333	.328	.323	.278	.822	0.93 (0.73–1.19)	.60	NS
*rs2738782* (A>G)	63677217	Intron 10	.474	.451	.460	.671	.024	.136	1.11 (0.89–1.39)	.33	NS
*rs2738783* (G>T)	63677259	Intron 10	.408	.484	.454	.241	.933	.355	0.72 (0.57–0.90)	**.005**	NS
*rs6010620*^a^ (A>G)	63678486	Intron 11	.320	.241	.273	.642	.964	.918	1.55 (1.19–2.01)	**.0009**	**.02**
*rs62207047* (C>T)	63679610	Intron 11	.126	.079	.098	.578	.628	.983	1.77 (1.20–2.59)	**.003**	NS
*rs3787089* (G>A)	63685277	Intron 13	.314	.239	.269	.284	.918	.520	1.55 (1.19–2.02)	**.001**	**.02**
*rs77086616* (C>T)	63685825	Exon 15 (T434M)	.012	.000	.005	2.71x10^-7^	-	1.55x10^-16^	-	**.01**^c^	**-**
*rs4809324*^a^ (T>C)	63686867	Intron 15	.127	.081	.099	.572	.720	.943	1.72 (1.17–2.51)	**.005**	NS
*rs79210260* (C>T)	63689576	Exon 22 (R651R)	.100	.101	.101	.702	.223	.234	1.01 (0.69–1.47)	.94	NS
*rs6062302*^a^ (T>C)	63689615	Exon 22 (D664D)	.349	.253	.290	.787	.561	.621	1.62 (1.26–2.10)	**.0002**	**.004**
*rs200933423* (C>G)	63694443	Exon 30 (L1022V)	.012	.007	.009	.848	.897	.824	1.99 (0.59–6.67)	.26	NS
*rs3208008*^a^ (A>C)	63694757	Exon 31 (Q1042H)	.369	.308	.333	.174	.918	.471	1.37 (1.07–1.76)	**.01**	NS
*rs115303435*^a^ (G>A)	63694806	Exon 31 (A1059T)	.066	.027	.042	.264	.596	.268	3.06 (1.69–5.54)	**.0002**	**.003**
*rs41309931* (G>T)	63695226	Intron 33	.192	.147	.165	.620	.700	.994	1.41 (1.04–1.91)	**.03**	NS
*rs2297441* (G>A)	63696229	3'UTR	.353	.295	.318	.255	.913	.562	1.36 (1.06–1.74)	**.01**	NS

Logistic regression analysis under additive model was used for calculating ORs and corresponding *P*-values for SNPs controlling age and sex as covariates.

Significant associations are shown in bold face.

^a^SNPs were analyzed in previous studies on glioma risk.

^b^Bonferroni-adjusted *P*-values by 22 SNP tests.

^c^The *P*-value was determined using the χ^2^ test because no variants were observed in PCs.

Abbreviation: Chr., chromosome; PC, population control; HWE, Hardy-Weinberg equilibrium; OR, odds ratio; CI, confidence interval; NS, not significant.

### Genetic effects of causal variants on glioma risk

**Fig 1:**
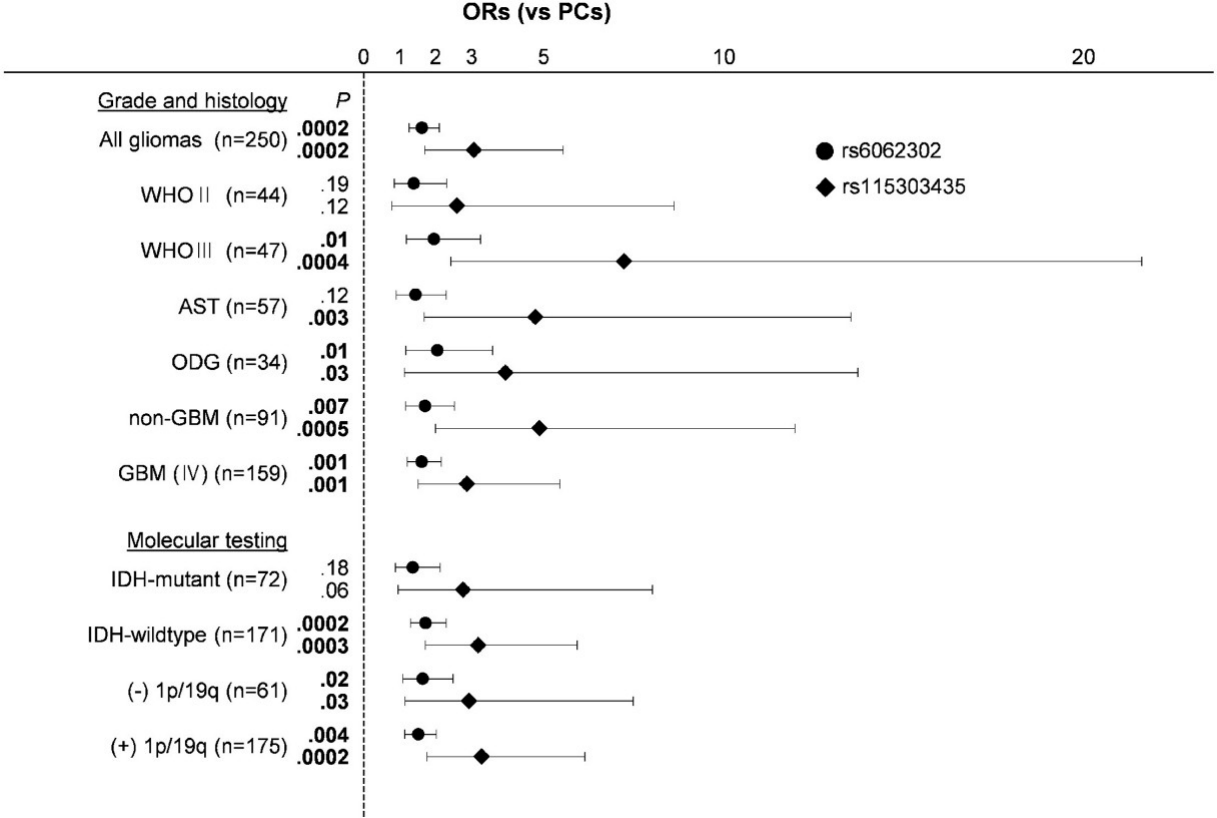
The association results of two independent SNPs between glioma subgroups and PCs. Logistic regression between glioma subgroups and PCs (n = 375) under additive model, adjusted by age and sex as covariates, was used for calculating ORs (95% CI) and P-values at rs6062302 (black round) and rs115303435 (black rhombus). The each plot indicates the point estimate of ORs on the x-axis shown with 95% CI on the error bars. Significant associations are bolded. Abbreviations: PC, population control; WHO, world health organization grade; AST, astrocytomas, ODG, oligodendrogliomas; GBM, glioblastomas; IDH-mutant, IDH1 or IDH2-mutated gliomas; IDH-wildtype, IDH-wildtype gliomas; (-) 1p/19q, 1p/19q codeletion; (+) 1p19q, 1p/19q non-codeletion; OR, odds ratio; CI, confidence interval.

To further confirm the independent association between significant SNPs and glioma risk, stepwise and conditional logistic regression analyses were conducted on the six significant *RTEL1* variants. As shown in Table 3, two SNPs (*rs6062302* and *rs115303435*) remained in the model at the parametric discriminant *P*-value (0.05). After applying conditional logistic regression, the same conclusion was independently reached, although *rs6062302* showed a much weaker association than *rs115303435* after the significant *RTEL1* variants were conditioned. In addition, we examined the differential association between the two independent SNPs (*rs6062302* and *rs115303435*) and glioma subgroups with respect to clinical characteristics such as WHO grade, histological type, and molecular alteration. They were found to be possibly involved with increased risk of gliomas regardless of histologic features, *IDH* mutations, and 1p/19q codeletion status (Fig 1).

**Table 3:** Independent association signals among glioma-associated *RTEL1* variants.

SNP	*P*	Stepwise *P*^a^	Conditional *P*-value by					
			*rs6089953*^b^	*rs3848669*	*rs6010620*^b^	*rs3787089*	*rs6062302*^b^	*rs115303435*
*rs6089953*^b^	**.001**	-	-	.93	.83	.58	.78	**.03**
*rs3848669*	**.0009**	-	.93	-	.59	.40	.93	**.02**
*rs6010620*^b^	**.0009**	-	.41	.63	-	.48	.45	**.02**
*rs3787089*	**.001**	-	.50	.70	.95	-	.19	**.03**
*rs6062302*^b^	**.0002**	**.0004**	**.05**	.07	**.05**	**.02**	-	**.004**
*rs115303435*	**.0002**	**.01**	**.006**	**.007**	**.006**	**.007**	**.01**	-

The *P*-values were obtained by logistic analysis between glioma patients (n = 250) and PCs (n = 375) under additive model.

Significant associations are shown in bold face.

^a^The significance level was set at 0.05 in stepwise selection of glioma-associated *RTEL1* SNPs.

^b^Previously identified loci in *RTEL1*.

### Assessment of the functional effects of *RTEL1* variants

Our significant missense variants were evaluated via *in silico* analysis using the TransFIC method. Although *rs115303435* (A1059T) had low functional and structural impact on the cancer data, *rs77086616* (T434M) was observed to have medium functional impact on *RTEL1* when the TransFIC method was applied with the SIFT tool. In the FuncPred method, a coding variant *rs6062302* (D664D) was predicted to alter transcription factor binding (S2 Table).

## Discussion

This study demonstrates that previously identified loci in *RTEL1* are confirmed to have an association with increased risk of adult gliomas. Moreover, two coding variants (*rs6062302* and *rs115303435*) were found to confer independent risk for glioma in *RTEL1*. A novel missense SNP (*rs77086616*, T434M) was also observed only in Korean glioma samples. In addition, we compared the associations between causal *RTEL1* markers and glioma subgroups based on grades of malignancy and histopathological subtypes.

A number of GWASs and candidate gene studies have identified *RTEL1* variants involved with genetic predispositions to glioma development [6, 7, 22]. A summary of studies between *RTEL1* variants and glioma risk is listed in S3 Table. Of the extensively investigated *RTEL1* SNPs, the intronic *rs6010620* was the most studied SNP in White and Chinese populations, although there were differences in the effect of this SNP on glioma risk [12]. Recently, the GWAS meta-analyses in up to 30,686 individuals showed that the significant association at *RTEL1 rs2297440* was observed between the risk of GBM and non-GBM tumors [7]. However, we did not find any association of the *RTEL1* variants from the case-only analysis (*rs2297440* was replaced by *rs6010620* with LD in East Asian populations).

In case-control studies, *rs6010620* and *rs2297440* were found to be associated with glioma development [11] and GBM risk [23] in Han Chinese populations. The intronic *rs6010620* and *rs4809324* were independent predictors of glioma risk in European ancestry individuals [9], whereas the risk of glioma development [11] and GBM [23] in Han Chinese was not influenced by *rs4809324*. *rs6010620*, *rs2297440*, and *rs4809324* were presented in one haplotype block which was associated with increased risk of astrocytoma in Chinese populations [24]. Unconditional logistic regression analysis showed that *rs6010620* and *rs4809324* were associated with increased risk of adult glioma in Korean populations (Table 2). The resulting LD between *rs6010620* and *rs4809324* was high (*r*^*2*^ = .94, S2 Fig).

In addition to intronic SNPs, the associations between the coding variants (*rs6062302* and *rs3208008*) and risk of adult gliomas were observed in US population study [10], a fact which was replicated in this study (S3 Table). An exome-wide association study has also identified a number of missense SNPs in *RTEL1*, including *rs3208008* (Q1042H) and *rs115303435* (A1059T), in a Han Chinese population, although *rs115303435* showed marginal association with GBM risk after conditional analysis by *rs6010620* (*P* = .059) [25]. Among those glioma-associated SNPs, *rs6062302* and *rs115303435* were replicated in our Korean subjects as causal SNPs associated with increased risk of adult gliomas (Table 3). In addition, the new glioma-specific variant (*rs77086616*, T434M) was identified only in certain cases and was found to be rare (MAF = .005) in this study. More than half of the low-frequency missense alleles were found to have deleterious effects with respect to the intensity of selective pressure among disease alleles [26]. Although *rs115303435* and *rs77086616* were only observed in this study and in East Asian populations [18], to date, little information has been confirmed between a population-specific variant and glioma risk.

Human RTEL1 is an essential DNA helicase which helps maintain genome stability through telomere maintenance and DNA repair [27]. However, few functional studies on RTEL1 with respect to glioma tumorigenesis have been conducted [22]. As such, it is difficult to assess the role of oncogenic or tumor suppressor pathways. Using *in silico* analysis, the splicing effect of *rs6062302* was studied (S2 Table). A rare variant (*rs77086616*, T434M) was found to have a medium impact on the RTEL1 function when the TransFIC method was applied. In addition, to identify the significant SNPs that alter the expression level of *RTEL1* in brain tissues, we searched for expression quantitative trait locus data (eQTL) from the UK Brain Expression Consosium (Braineac; http://www.braineac.org). Healthy individuals of Western European descent with the *rs6062302* C>T allele showed increased mRNA expression levels in the temporal and frontal cortex compared to other brain regions. However, *rs77086616* and *rs115303435* data were not available in the public eQTL database.

Recent advances in molecular profiling have contributed to understanding the molecular aberrations of diffuse gliomas in adults [28]. *IDH* mutations, most of the R132 codon of *IDH1*, are found frequently in a majority of astrocytomas and oligodendrogliomas cases [16]. The combined chromosome imbalances of 1p and 19q resulting in loss of heterozygosity are prognostic biomarkers for oligodendroglial tumors [15]. Likewise, our oligodendrogliomas were shared in almost all cases with both *IDH* mutations and 1p/19q codeletion (Table 1). Among the associated inherited risk variants, *RTEL1* variants were found to be associated with risk of glioma regardless of molecular alterations [6, 14]. The similar pattern was observed for our causal variants with glioma, stratified by 2016 CNS WHO, morphology, *IDH* mutations, and 1p/19q codeletion (Fig 1). In particular, *rs6062302* and *rs115303435* were more significantly associated with *IDH*-wildtype gliomas than with *IDH* mutants. The ORs of *rs115303435* were higher than *rs6062302* in all glioma classes. However, the subgroup analyses were limited in their statistical power due to small number of cases analyzed; these results should be interpreted with caution. In addition, we used general PCs matched for age and sex to estimate the impact of the association between *RTEL1* variants and risk of adult gliomas. Although population-based controls lack clinical information for detailed inclusion and exclusion criteria, the use of PCs in this study may be considered as an alternative method for assessing genetic effects [29].

Despite these limitations, the present study reinforces understanding of *RTEL1* association with adult gliomas in Korean populations. Future studies considering our findings in larger glioma samples with molecular alterations must test the reproducible markers for understanding of glioma pathogenesis.

## Supporting information

S1 Table Comparisons of *IDH* mutations and 1p/19q co-deletion status in each study group. Abbreviation: US indicates United States; GBM, glioblastoma.
(DOCX)

S2 Table *In silico* analysis of *RTEL1 rs6062302* (D664D). *In silico* analysis was conducted using FuncPred (https://snpinfo.niehs.nih.gov/snpinfo/snpfunc.html). Lowercase alleles in motif (forward strand) indicate the *rs6062302* position. Threshold score for associated splicing factor SF2 /ASF was 1.956. Abbreviation: ESE, exonic splicing enhancer; ESS, exonic splicing silencer.
(DOCX)

S3 Table The Studies between *RTEL1* variants and glioma risk. Significant associations are shown in bold face. Abbreviation: OR (95% CI), odds ratio (95% confidence interval); NA, not available. *Causal SNPs on glioma risk in present study ^§^Protective allele
(DOCX)

S1 Fig The gene map of *RTEL1* (NM_032957.4) on chromosome 20q13.33 (38.444 kb). (A) A map of *RTEL1*. Coding exons are marked by black blocks, and 5’- and 3’-untranslated regions by white blocks. (B) Haplotypes of *RTEL1*. The BL1_*ht4* (OR = 1.67, *P* = .003), BL2_*ht1* (OR = 0.70, *P* = .003), BL2_*ht4* (OR = 1.86, *P* = .002), BL3_*ht1* (OR = 1.40, *P* = .007), and BL3_*ht2* (OR = 1.67, *P* = .03) were associated with risk of adult gliomas. Abbreviation: ht, haplotype; PC, population control.
(TIF)

S2 Fig The LD structure of genotyped *RTEL1* SNPs. The region includes three LD blocks marked by triangles with black lines. The SNPs in the haplotype blocks are shown in bold. Numbers and grayscale shades in boxes indicate *r*^*2*^ values.
(TIF)
