# Supplementary material for: Association analysis of RTEL1 variants with risk of adult gliomas in a Korean population
Source: PLoS One. 2018 Nov 21;13(11):e0207660. doi: 10.1371/journal.pone.0207660 (PMC6248978; doi:10.1371/journal.pone.0207660)
Supplement: S1 Table — Abbreviation: US indicates United States; GBM, glioblastoma. (DOCX) [file pone.0207660.s001.docx]

| Study name | Country | WHO grading | Total (n) | *IDH1* or *IDH2* mutation | 1p/19q codeletion |
| --- | --- | --- | --- | --- | --- |
| Present study | Korea | Ⅱ-Ⅳ | 250 | 72/243 (29.6%) | 61/236 (25.8%) |
|  |  | non-GBM (Ⅱ-Ⅲ) | 91 | 55/87 (63.2%) | 44/89 (49.4%) |
|  |  | GBM (Ⅳ) | 159 | 17/156 (10.9%) | 17/147 (11.6%) |
|  |  |  |  |  |  |
| Mukasa 2012 ^[^[^1^](#_ENREF_1)^]^ | Japan | Ⅱ-Ⅳ | 235 | 75/235 (31.9%) | 36/235 (15.3%) |
|  |  | non-GBM (Ⅱ-Ⅲ) | 110 | 62/110 (56.4%) | 34/110 (30.9%) |
|  |  | GBM (Ⅳ) | 125 | 13/125 (10.4%) | 2/125 (1.6%) |
|  |  |  |  |  |  |
| Di Stefano 2013 ^[^[^2^](#_ENREF_2)^]^ | France | Ⅱ-Ⅳ | 1372 | 375/916 (40.9%) | 137/896 (15.3%) |
|  |  | non-GBM (Ⅱ-Ⅲ) | 921 | 344/610 (56.4%) | 133/589 (22.6%) |
|  |  | GBM (Ⅳ) | 451 | 31/306 (10.1%) | 4/307 (1.3%) |
|  |  |  |  |  |  |
| Rice 2013 ^[^[^3^](#_ENREF_3)^]^ | US | Ⅱ-Ⅳ | 1102 | 34.0% |  |
|  |  | non-GBM (Ⅱ-Ⅲ) | 439 | 73.5% |  |
|  |  | GBM (Ⅳ) | 663 | 8.0% |  |
|  |  |  |  |  |  |
| Killela 2014 ^[^[^4^](#_ENREF_4)^]^ | US | Ⅱ-Ⅳ | 473 | 227/473 (48.0%) | 61/473 (12.9%) |
|  |  | non-GBM (Ⅱ-Ⅲ) | 233 | 203/233 (87.1%) | 61/233 (26.2%) |
|  |  | GBM (Ⅳ) | 240 | 24/240 (10.0%) | 0/240 |
|  |  |  |  |  |  |
| Eckel‑Passow 2015 ^[^[^5^](#_ENREF_5)^]^ | US | Ⅱ-Ⅳ | 1087 | 532/1086 (49.0%) |  |

**References**

1. Mukasa A, Takayanagi S, Saito K, Shibahara J, Tabei Y, Furuya K, et al. Significance of IDH mutations varies with tumor histology, grade, and genetics in Japanese glioma patients. Cancer science. 2012;103(3):587-92. doi: 10.1111/j.1349-7006.2011.02175.x. PubMed PMID: 22136423.

2. Di Stefano AL, Enciso-Mora V, Marie Y, Desestret V, Labussiere M, Boisselier B, et al. Association between glioma susceptibility loci and tumour pathology defines specific molecular etiologies. Neuro-oncology. 2013;15(5):542-7. doi: 10.1093/neuonc/nos284. PubMed PMID: 23161787; PubMed Central PMCID: PMC3635509.

3. Rice T, Zheng S, Decker PA, Walsh KM, Bracci P, Xiao Y, et al. Inherited variant on chromosome 11q23 increases susceptibility to IDH-mutated but not IDH-normal gliomas regardless of grade or histology. Neuro-oncology. 2013;15(5):535-41. doi: 10.1093/neuonc/nos324. PubMed PMID: 23361564; PubMed Central PMCID: PMC3635511.

4. Killela PJ, Pirozzi CJ, Healy P, Reitman ZJ, Lipp E, Rasheed BA, et al. Mutations in IDH1, IDH2, and in the TERT promoter define clinically distinct subgroups of adult malignant gliomas. Oncotarget. 2014;5(6):1515-25. doi: 10.18632/oncotarget.1765. PubMed PMID: 24722048; PubMed Central PMCID: PMC4039228.

5. Eckel-Passow JE, Lachance DH, Molinaro AM, Walsh KM, Decker PA, Sicotte H, et al. Glioma Groups Based on 1p/19q, IDH, and TERT Promoter Mutations in Tumors. The New England journal of medicine. 2015;372(26):2499-508. doi: 10.1056/NEJMoa1407279. PubMed PMID: 26061753; PubMed Central PMCID: PMC4489704.
