# Supplementary material for: Association analysis of RTEL1 variants with risk of adult gliomas in a Korean population
Source: PLoS One. 2018 Nov 21;13(11):e0207660. doi: 10.1371/journal.pone.0207660 (PMC6248978; doi:10.1371/journal.pone.0207660)
Supplement: S2 Table — In silico analysis was conducted using FuncPred (https://snpinfo.niehs.nih.gov/snpinfo/snpfunc.html). Lowercase alleles in motif (forward strand) indicate the rs6062302 position. Threshold score for associated splicing factor SF2 /ASF was 1.956. Abbreviation: ESE, exonic splicing enhancer; ESS, exonic splicing silencer. (DOCX) [file pone.0207660.s002.docx]

| Allele | Motif | Associated splicing factor | Score | Method |  | Splicing (abolish domain) | Conservation score |
| --- | --- | --- | --- | --- | --- | --- | --- |
| C | TGGAcGA | SF2/ASF2 | 2.22 | ESEfind |  | Y | 1.000 |
|  | TGGAcGA | SF2/ASF1 | 2.94 | ESEfind |  |  |  |
| T | AtGAGA | - | - | RESCUE-ESE |  |  |  |
|  | GAtGAG | - | - | RESCUE-ESE |  |  |  |
