# Supplementary material for: Association analysis of RTEL1 variants with risk of adult gliomas in a Korean population
Source: PLoS One. 2018 Nov 21;13(11):e0207660. doi: 10.1371/journal.pone.0207660 (PMC6248978; doi:10.1371/journal.pone.0207660)
Supplement: S3 Table — Significant associations are shown in bold face. Abbreviation: OR (95% CI), odds ratio (95% confidence interval); NA, not available. *Causal SNPs on glioma risk in present study §Protective allele (DOCX) [file pone.0207660.s003.docx]

| SNP | Study name | Case/control | Ethnicity | WHO grading | Risk allele | OR (95% CI) | *P* | Conditional *P* on *rs6010620* | Genetic model |
| --- | --- | --- | --- | --- | --- | --- | --- | --- | --- |
| *rs6089953* | Present study | 250/375 | East Asian | Ⅱ-Ⅳ | G | 1.52 (1.17-1.97) | **.001** | .83 | Additive |
|  | Egan 2011 ^[^[^1^](#_ENREF_1)^]^ | 639/649 | - | All | A^§^ | 0.73 (0.61-0.88) | **.001** | NA | Allelic |
|  | Wrensch 2009 ^[^[^2^](#_ENREF_2)^]^ | 865/4,086 | White | Ⅲ-Ⅳ | A^§^ | 0.65 (0.57-0.75) | **<.0001** | NA | Mantel-Haenszel |
|  |  |  |  |  |  |  |  |  |  |
| *rs6010620* | Present study | 250/375 | East Asian | Ⅱ-Ⅳ | G | 1.55 (1.19-2.01) | **.0009** | - | Additive |
|  | Melin 2017 ^[^[^3^](#_ENREF_3)^]^ | 12,496/18,190 | White | All | G | 1.34 (1.29-1.40) | **<.0001** | - | Additive |
|  | Zhao 2017 ^[^[^4^](#_ENREF_4)^]^ | 1,038/1,008 | East Asian | Ⅳ | G | 1.43 (1.25-1.64) | **<.0001** | - | Additive |
|  | Walsh 2014 ^[^[^5^](#_ENREF_5)^]^ | 1,644/7,736 | White | Ⅲ-Ⅳ | G | 1.56 (1.42-1.72) | **<.0001** | - | Additive |
|  | Li 2013 ^[^[^6^](#_ENREF_6)^]^ | 629/645 | East Asian | All | G | 1.32 (1.11-1.57) | **.0015** | - | Additive |
|  | Egan 2011 ^[^[^1^](#_ENREF_1)^]^ | 639/649 | - | All | A^§^ | 0.72 (0.60-0.87) | **.001** | - | Allelic |
|  | Wrensch 2009 ^[^[^2^](#_ENREF_2)^]^ | 867/4,165 | White | Ⅲ-Ⅳ | A^§^ | 0.66 (0.57-0.76) | **<.0001** | - | Mantel-Haenszel |
|  | Shete 2009 ^[^[^7^](#_ENREF_7)^]^ | 2,545/2,953 | White | All | G | 1.28 (1.21-1.35) | **<.0001** | - | Additive |
|  |  |  |  |  |  |  |  |  |  |
| *rs4809324* | Present study | 250/375 | East Asian | Ⅱ-Ⅳ | C | 1.72 (1.17-2.51) | **.005** | .22 | Additive |
|  | Zhao 2017 ^[^[^4^](#_ENREF_4)^]^ | 1,038/1,008 | East Asian | Ⅳ | C | 1.35 (1.12-1.64) | **.002** | .77 | Additive |
|  | Wrensch 2009 ^[^[^2^](#_ENREF_2)^]^ | 868/4,153 | White | Ⅲ-Ⅳ | C | 1.60 (1.37-1.87) | **<.0001** | NA | Mantel-Haenszel |
|  |  |  |  |  |  |  |  |  |  |
| *rs6062302** | Present study | 250/375 | East Asian | Ⅱ-Ⅳ | C | 1.62 (1.26-2.10) | **.0002** | **.05** | Additive |
|  | Egan 2011 ^[^[^1^](#_ENREF_1)^]^ | 639/649 | - | All | T^§^ | 0.71 (0.58-0.86) | **.0004** | NA | Allelic |
|  |  |  |  |  |  |  |  |  |  |
| *rs3208008* | Present study | 250/375 | East Asian | Ⅱ-Ⅳ | C | 1.37 (1.07-1.76) | **.01** | .84 | Additive |
|  | Zhao 2017 ^[^[^4^](#_ENREF_4)^]^ | 1,038/1,008 | East Asian | Ⅳ | C | 1.36 (1.20-1.55) | **<.0001** | .23 | Additive |
|  | Egan 2011 ^[^[^1^](#_ENREF_1)^]^ | 639/649 | - | All | A^§^ | 0.70 (0.57-0.85) | **.0003** | NA | Allelic |
|  |  |  |  |  |  |  |  |  |  |
| *rs115303435** | Present study | 250/375 | East Asian | Ⅱ-Ⅳ | A | 3.06 (1.69-5.54) | **.0002** | **.006** | Additive |
|  | Zhao 2017 ^[^[^4^](#_ENREF_4)^]^ | 1,038/1,008 | East Asian | Ⅳ | A | 1.71 (1.21-2.42) | **.002** | .06 | Additive |
|  |  |  |  |  |  |  |  |  |  |
| *rs2297440* | Melin 2017 ^[^[^3^](#_ENREF_3)^]^ | 12,496/18,190 | White | All | C | 1.36 (1.30-1.42) | **<.0001** | NA | Additive |
|  | Li 2013 ^[^[^6^](#_ENREF_6)^]^ | 629/645 | East Asian | All | C | 1.34 (1.12-1.59) | **.001** | NA | Additive |
|  | Egan 2011 ^[^[^1^](#_ENREF_1)^]^ | 639/649 | - | All | T^§^ | 0.73 (0.60-0.89) | **.002** | NA | Allelic |
|  | Shete 2009 ^[^[^7^](#_ENREF_7)^]^ | 2,545/2,953 | White | All | C | 1.27 (1.20-1.34) | **<.0001** | NA | Additive |

**References**

1. Egan KM, Thompson RC, Nabors LB, Olson JJ, Brat DJ, Larocca RV, et al. Cancer susceptibility variants and the risk of adult glioma in a US case-control study. Journal of neuro-oncology. 2011;104(2):535-42. doi: 10.1007/s11060-010-0506-0. PubMed PMID: 21203894; PubMed Central PMCID: PMC3138895.

2. Wrensch M, Jenkins RB, Chang JS, Yeh RF, Xiao Y, Decker PA, et al. Variants in the CDKN2B and RTEL1 regions are associated with high-grade glioma susceptibility. Nature genetics. 2009;41(8):905-8. doi: 10.1038/ng.408. PubMed PMID: 19578366; PubMed Central PMCID: PMC2923561.

3. Melin BS, Barnholtz-Sloan JS, Wrensch MR, Johansen C, Il'yasova D, Kinnersley B, et al. Genome-wide association study of glioma subtypes identifies specific differences in genetic susceptibility to glioblastoma and non-glioblastoma tumors. Nature genetics. 2017;49(5):789-94. doi: 10.1038/ng.3823. PubMed PMID: 28346443; PubMed Central PMCID: PMC5558246.

4. Zhao Y, Yun D, Zou X, Jiang T, Li G, Hu L, et al. Whole exome-wide association study identifies a missense variant in SLC2A4RG associated with glioblastoma risk. American journal of cancer research. 2017;7(9):1937-47. PubMed PMID: 28979815; PubMed Central PMCID: PMC5622227.

5. Walsh KM, Codd V, Smirnov IV, Rice T, Decker PA, Hansen HM, et al. Variants near TERT and TERC influencing telomere length are associated with high-grade glioma risk. Nature genetics. 2014;46(7):731-5. doi: 10.1038/ng.3004. PubMed PMID: 24908248; PubMed Central PMCID: PMC4074274.

6. Li G, Jin T, Liang H, Zhang Z, He S, Tu Y, et al. RTEL1 tagging SNPs and haplotypes were associated with glioma development. Diagnostic pathology. 2013;8:83. doi: 10.1186/1746-1596-8-83. PubMed PMID: 23683922; PubMed Central PMCID: PMC3661361.

7. Shete S, Hosking FJ, Robertson LB, Dobbins SE, Sanson M, Malmer B, et al. Genome-wide association study identifies five susceptibility loci for glioma. Nature genetics. 2009;41(8):899-904. doi: 10.1038/ng.407. PubMed PMID: 19578367; PubMed Central PMCID: PMC4501476.
