# Supplementary figures and images for: Association analysis of RTEL1 variants with risk of adult gliomas in a Korean population
Source: PLoS One. 2018 Nov 21;13(11):e0207660. doi: 10.1371/journal.pone.0207660 (PMC6248978; doi:10.1371/journal.pone.0207660)

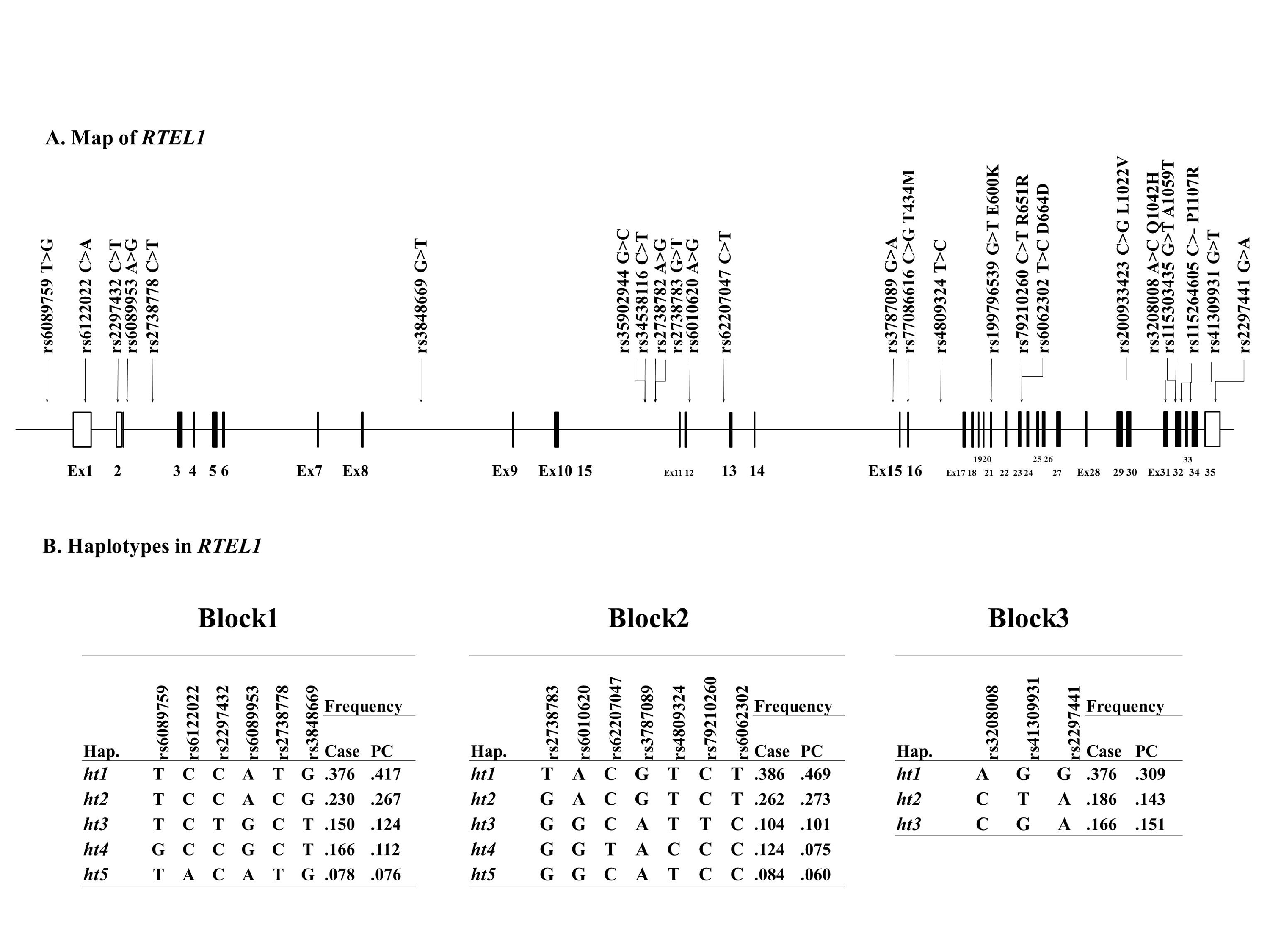

Supplement: S1 Fig — (A) A map of RTEL1. Coding exons are marked by black blocks, and 5’- and 3’-untranslated regions by white blocks. (B) Haplotypes of RTEL1. The BL1_ht4 (OR = 1.67, P = .003), BL2_ht1 (OR = 0.70, P = .003), BL2_ht4 (OR = 1.86, P = .002), BL3_ht1 (OR = 1.40, P = .007), and BL3_ht2 (OR = 1.67, P = .03) were associated with risk of adult gliomas. Abbreviation: ht, haplotype; PC, population control. (TIF) [file pone.0207660.s004.tif]

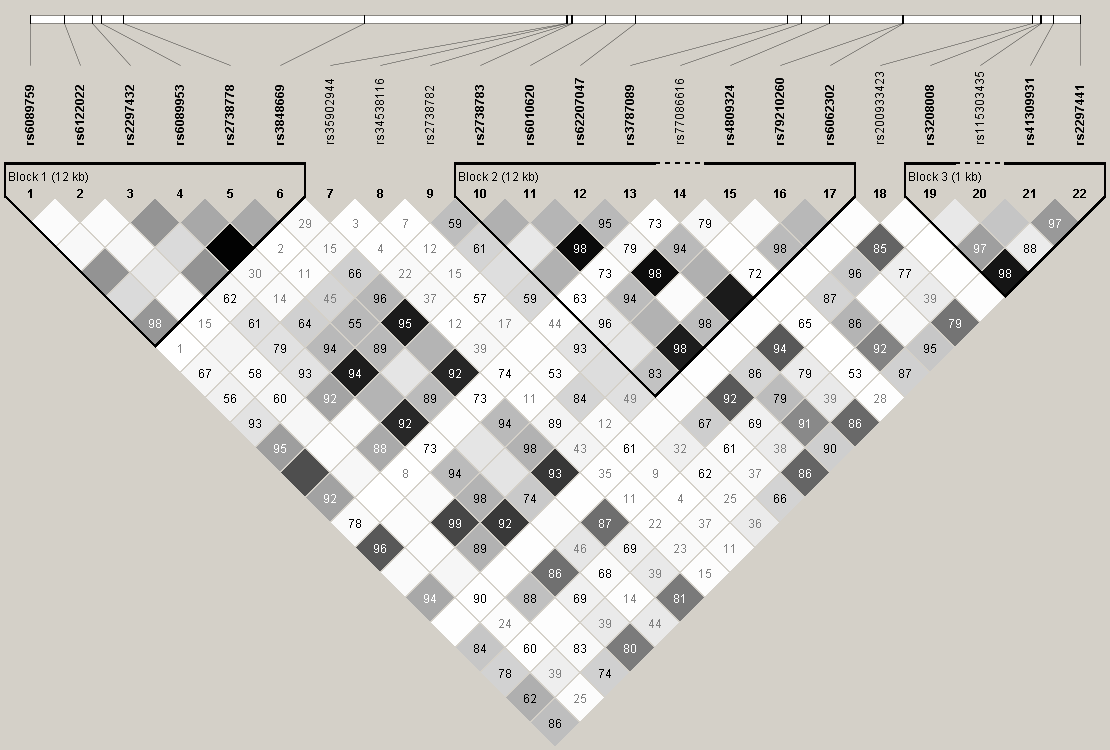

Supplement: S2 Fig — The region includes three LD blocks marked by triangles with black lines. The SNPs in the haplotype blocks are shown in bold. Numbers and grayscale shades in boxes indicate r2 values. (TIF) [file pone.0207660.s005.tif]
